# Supplementary material for: TUG protein acts through a disordered region to organize the early secretory pathway
Source: Nat Commun. 2025 Jul 1;16:5518. doi: 10.1038/s41467-025-60691-8 (PMC12218103; doi:10.1038/s41467-025-60691-8)
Supplement: Supplementary file 1 — Supplementary Information [file 41467_2025_60691_MOESM1_ESM.pdf]

Supplementary Data

TUG protein acts through a disordered region to organize the early secretory pathway

Anup Parchure[1,2]*, Helen Tejada[1], Zhiqun Xi[2], Yeongho Kim[2], Maohan Su[2], You Yan[3], Omar Julca-Zevallos[1,4], Abel R. Alcázar-Román[1,5], Marie Villemeur[6], Xinran Liu[2], Derek Toomre[2], Ishier Raote[6] and Jonathan S. Bogan[1,2,7]*

[1]Section of Endocrinology and Metabolism, Department of Internal Medicine, Yale School of Medicine, New Haven, CT 06520-8020, USA.
[2]Department of Cell Biology, Yale School of Medicine, New Haven, CT 06520, USA.
[3]Independent researcher
[4]Present address: Evolution Health Group, LLC, New York, NY, USA
[5]Present address: Eukaryotic Microbiology, Institute of Functional Microbial Genomics, Heinrich-Heine-University, 40225 Düsseldorf, Germany.
[6] Université Paris Cité, CNRS UMR7592, Institut Jacques Monod, F-75013 Paris, France
[7]Yale Center for Molecular and Systems Metabolism, Yale School of Medicine, New Haven, CT 06520, USA.

*Correspondence: anupparchure@gmail.com or jonathan.bogan@yale.edu

This PDF includes:
Supplementary Figures 1 to 9

Supplementary Figure 1

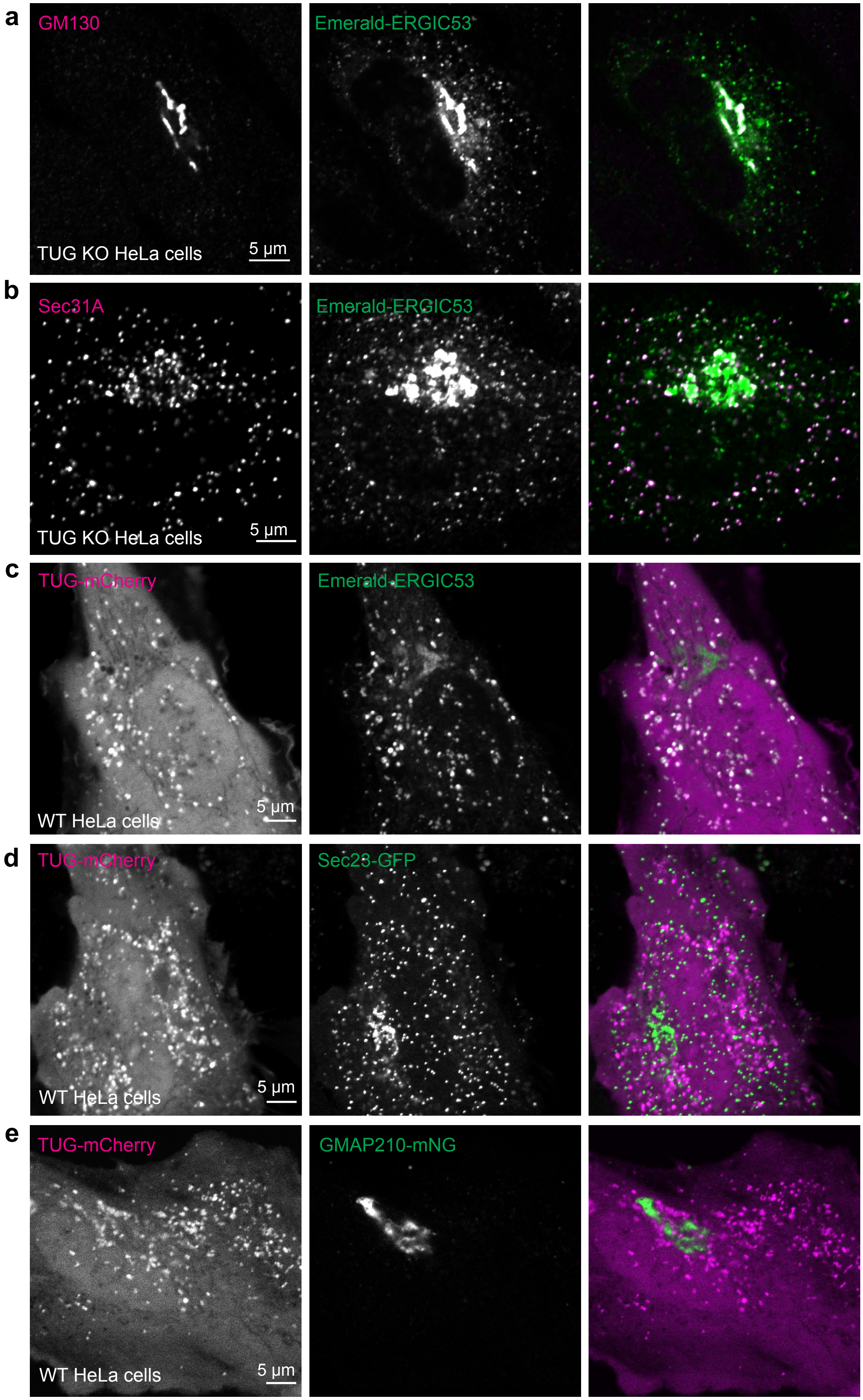

Supplementary Fig. 1 | Localization of Em-ERGIC53 and TUG-mCherry in HeLa cells
a, b) Images of TUG KO HeLa cells transfected with Em-ERGIC53 and fixed and stained with GFP nanobody (green) to amplify the ERGIC53 signal and with antibodies against GM130 (magenta; b) or Sec31 (magenta; c) to observe the localization of these proteins at the Golgi and at ERES, respectively. **c-e)** Images from WT HeLa cells co-transfected with Tug-mCherry (magenta) and Em-ERGIC53 (green; c), Sec23-GFP (green; d) and monomeric Neon Green (mNG) -tagged GMAP210 (green; e). Similar to the results obtained from TUG KO HeLa cells, TUG-mCherry extensively colocalizes with punctate ERGIC53 positive structures but does not overlap with Sec23 labeled structures and is excluded from the Golgi apparatus. Experiments were performed twice with similar results.

Supplementary Figure 2

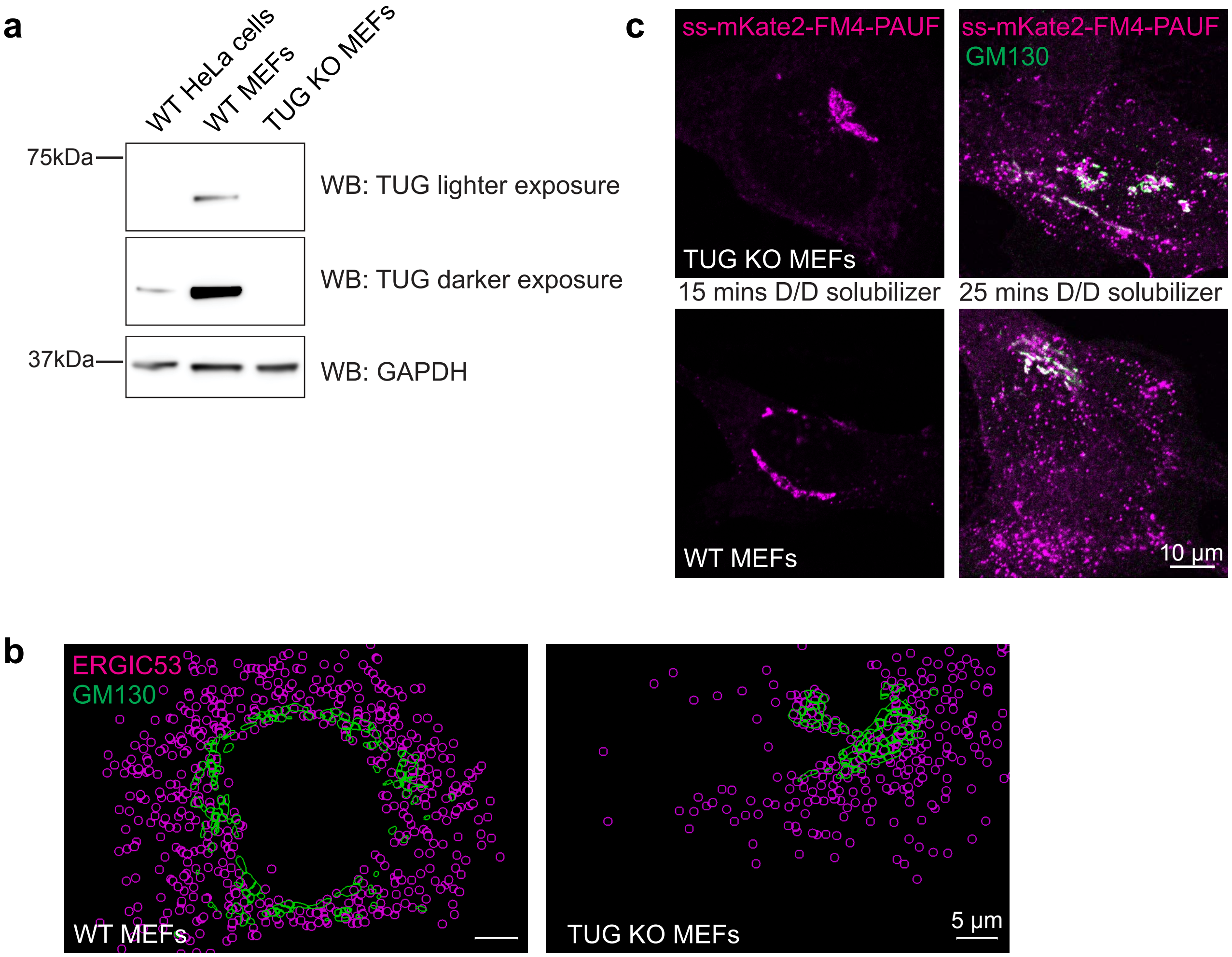

Supplementary Fig. 2 | Supporting data for ERGIC localization and anterograde trafficking studies

a) Western blot probed using anti-TUG antibody to monitor the relative abundance of TUG protein in WT HeLa cells and WT MEFs, as well as in TUG KO MEFs. In lighter exposure (top), the band is only visible in WT MEFs. However, upon darker exposure (middle), the band is visualized in WT HeLa cells, but no signal is seen from TUG KO MEFs. The bottom panel represents probing the same blot using anti-GAPDH antibody as a loading control. From the western blot, it is evident that HeLa cells express significantly lower levels of TUG abundance, compared to WT MEFs. There is a complete absence of protein expression in TUG KO MEFs, as expected. Experiments were performed at least twice with similar results. **b)** Representative surfaces from WT and TUG KO MEFs to denote ERGIC53 structures (magenta) and the Golgi, marked by GM130 (green). The surfaces are at the center of the outlines used and are from a 1-micron depth in Z. **c)** Images of ss-mKate2- FM4-PAUF are shown at 15 and at 25 min. after addition of D/D solubilizer. In the images taken at 15 min., most PAUF is present at the Golgi, both in TUG KO MEFs and in WT control MEFs. At 25 min., PAUF is present in post-Golgi vesicles in both cell lines. Data are representative of three experiments for 15 min. timepoint and two experiments for the 25 min. timepoint.

Supplementary Figure 3

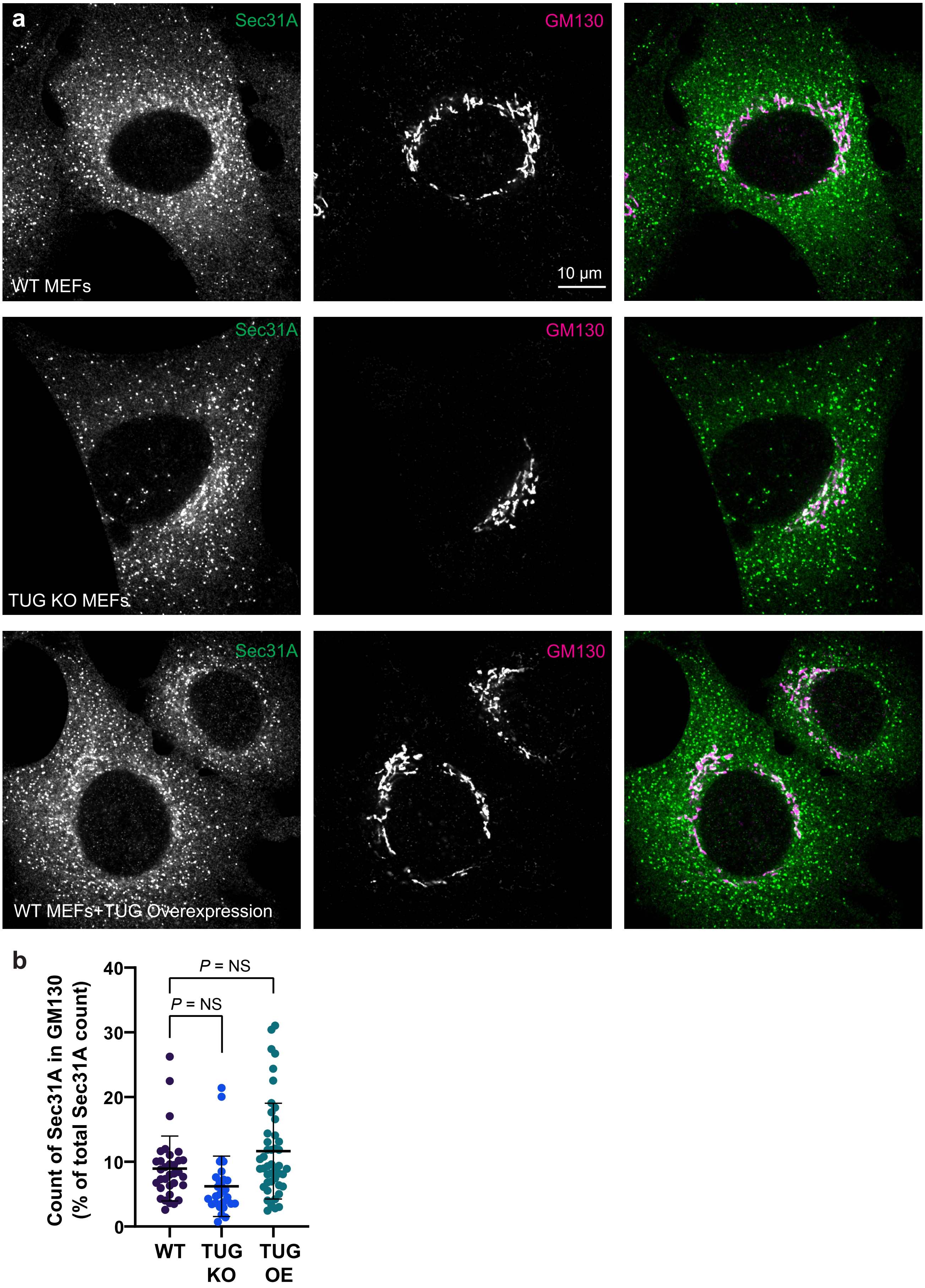

Supplementary Fig. 3 | ERES localization in relation to the *cis*-Golgi
a) Images which are a single plane from a confocal stack from WT MEFs (top), TUG KO MEFs (middle) and MEFs overexpressing untagged TUG (bottom) were fixed and stained using antibodies against GM130 (magenta) and Sec31 (green) to monitor the distribution of ERES in relation to the cis-Golgi. **b)** The graph quantifies the percentage of ERES that colocalize with GM130 per cell in each of the conditions. N=33 WT cells, 30 TUG KO cells, and 45 TUG overexpressing (OE) cells were used in the analysis. No significant difference was observed between the WT and either TUG KO or overexpressing cells.

Supplementary Figure 4

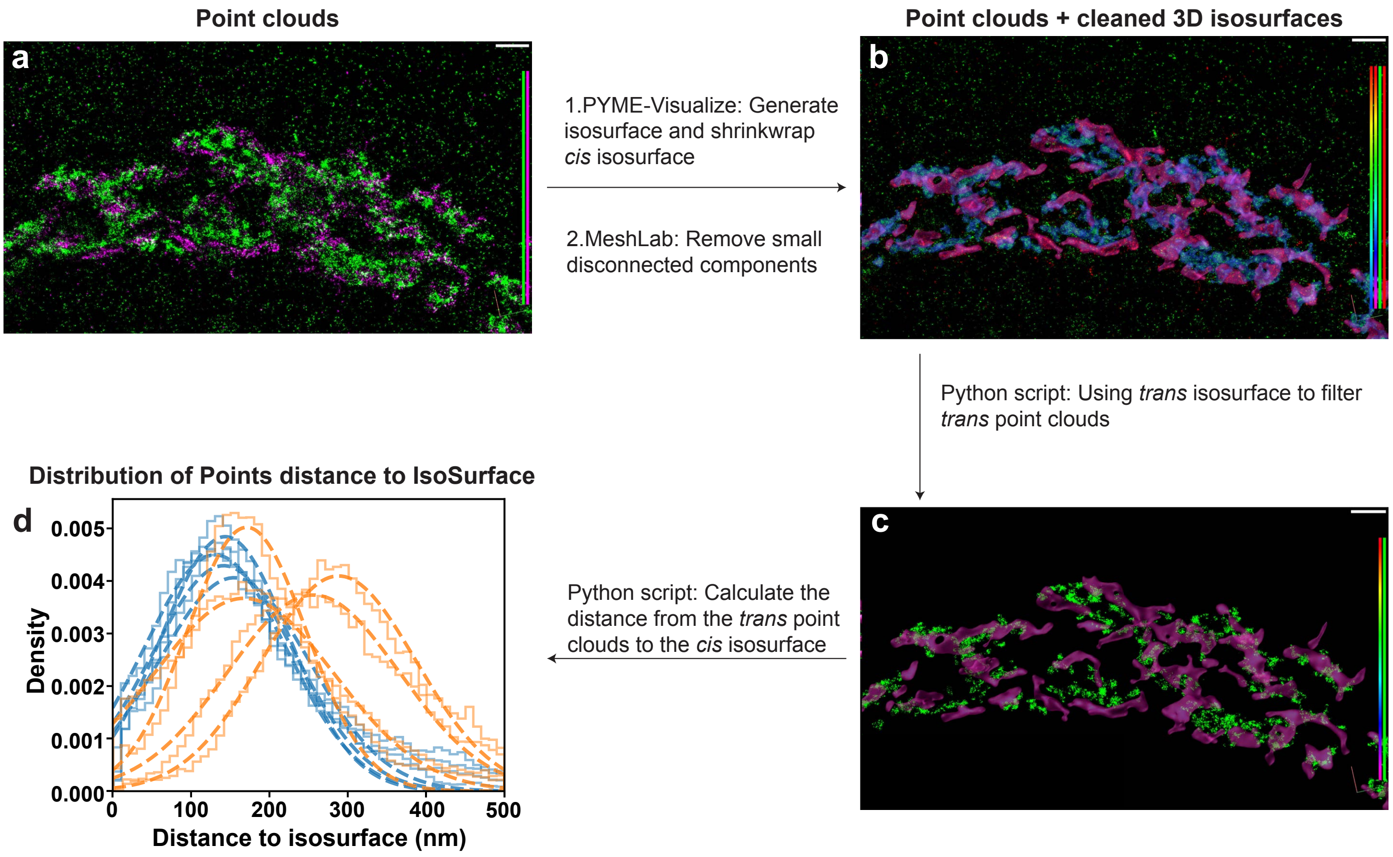

Supplementary Fig. 4 | Methods to analyze distance from cis- to trans- Golgi cisterna
a) MEF cells imaged by 4Pi-SMS visualized as point clouds. Magenta: GM130 (cis); green: Golgin97 + p230 (trans). Scale bar: 1 μm. **b)** Isosurfaces generated by PYME-Visualize. Magenta isosurface encloses GM130 points and blue isosurface encloses Golgin97 + p230 points. The cis isosurface has been processed with shrinkwrapping in PYME-Visualize, and both meshes have been cleaned up to remove noise. **c)** Cis isosurface and filtered trans points only are shown. **d)** Histograms of the distances of trans points (labeled by Golgin97 and p230) to the cis (labeled by GM130) isosurface for four WT (blue stepped lines) and TUG KO (orange stepped lines) cells. Gaussian curves (dotted) were fitted in each histogram. The peak values for the histograms in WT MEFs are 154.9 nm, 130.6 nm, 141.6 nm and 143.5 nm, while those for the TUG KO MEFs are 171.1 nm, 167.5 nm, 256.0 nm and 288.3 nm.

Supplementary Figure 5

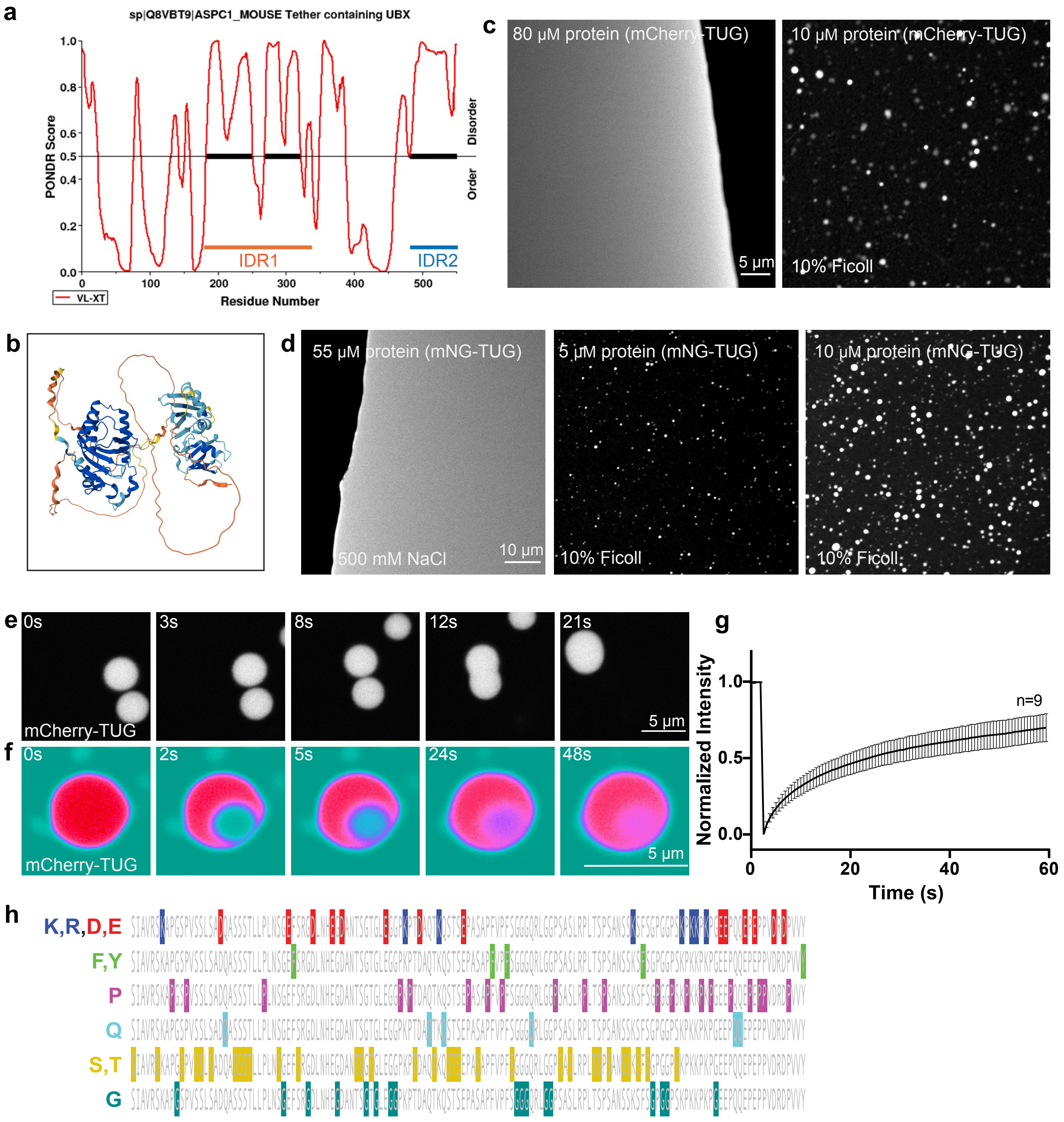

Supplementary Fig. 5 | Purified TUG protein forms condensates *in vitro*
a) Predictions of disordered regions in murine TUG protein were generated using PONDR. TUG protein is characterized by the presence of an internal disordered region (IDR1, residues 183-321, orange) and a smaller disordered region at the C-terminus (IDR2, residues 462-550, blue), when analyzed using the VL-XT algorithm. **b)** Alpha fold prediction of the murine TUG protein also shows the presence of disordered regions, interspersed between structured regions. **c)** Purified mCherry tagged TUG protein (80 µM) does not form condensates in phase separation buffer containing 125 mM NaCl (left). Yet, in presence of 10% Ficoll 400, condensates are seen at 10 µM protein concentration. **d)** Purified mNG tagged TUG does not form condensates in presence of buffer containing 500 mM NaCl. However, at physiological salt concentrations, protein forms condensates in a concentration dependent manner in presence of 10% Ficoll 400. **e)** A set of images extracted from time lapse imaging demonstrate the coalescence of two mCherry-TUG condensates in proximity, followed by relaxation to a spherical shape, which is reflective of a liquid-like behavior. **f)** A set of images extracted from time lapse imaging of a mCherry-TUG condensate to monitor fluorescence recovery post photobleaching. Images are pseudocolored to show changes in fluorescence intensity. **g)** Quantification of normalized intensity as a function of time before and after photobleaching a small region within a condensate. There is close to 70% recovery within a minute. Data N=9 condensates are represented as mean ± s.d. **h)** The distribution of particular amino acids within the TUG IDR1 domain is indicated. Amino acids are indicated at left, and their positions in the IDR1 sequence is shown by colored vertical bars.

Supplementary Figure 6

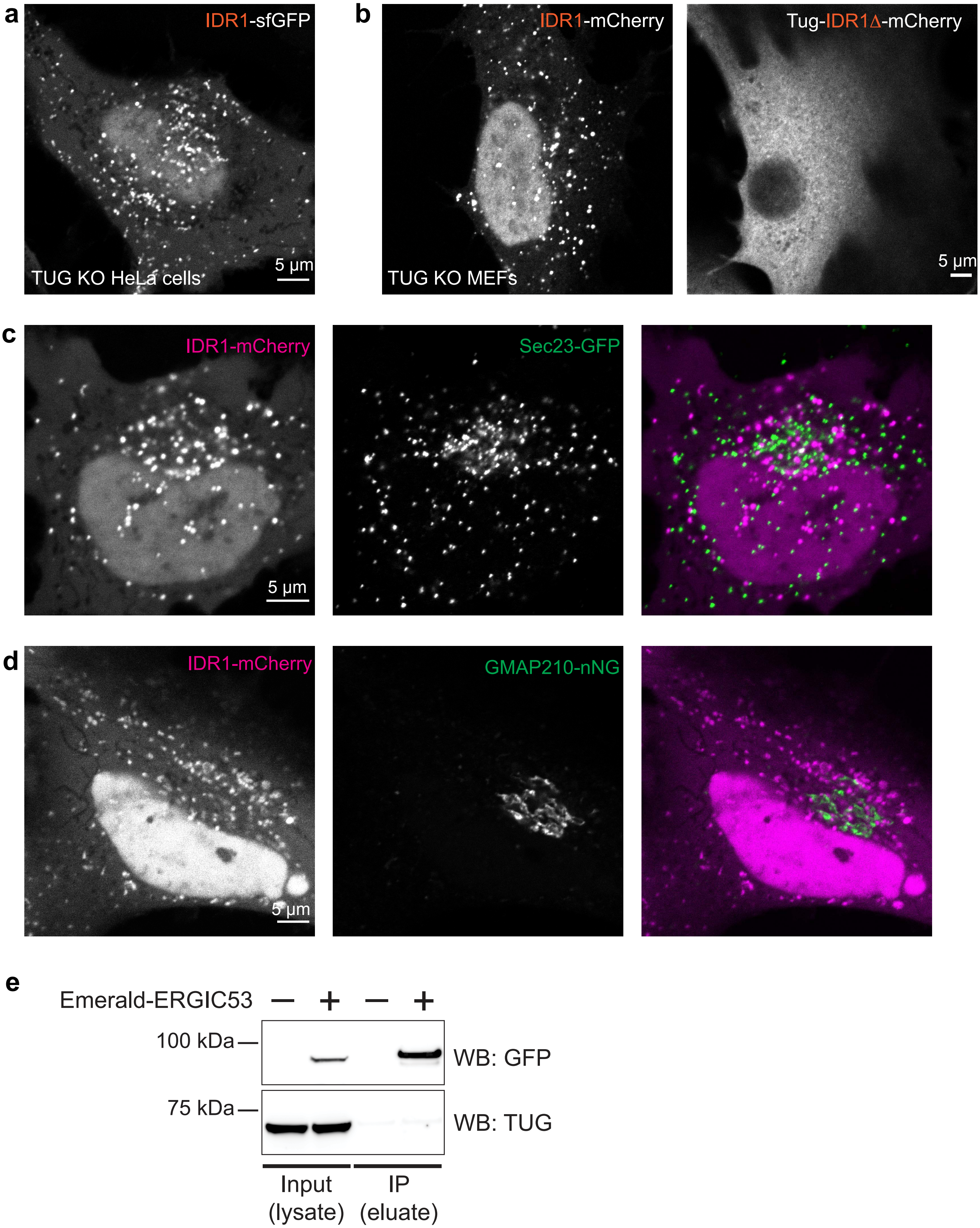

Supplementary Fig. 6 | TUG IDR1 is necessary and sufficient for ERGIC localization
a) Images from TUG KO HeLa cells transfected with IDR1 tagged with monomeric superfolder GFP (IDR1-sfGFP) show that IDR1 distribution is similar to that observed upon expression of the IDR1-mCherry construct (see Fig. 3b). This indicates that the distribution of the IDR1 in cells is independent of the fluorescent tag or the linker sequence, and that it is inherent in the protein sequence. Two independent experiments showed similar results. **b)** Images of TUG KO MEFs infected with retroviruses to express IDR1-mCherry (left) or Tug-IDR1Δ-mCherry (right). Cells were images 48 hours after the infection. Three independent infections were carried out. Similar to the distribution of these proteins in TUG KO HeLa cells (see Fig. 3b, c), expression of IDR1 alone in MEFs results in a punctate distribution, while the deletion of the IDR1 results in a diffuse distribution and exclusion of the fluorescent signal from the nucleus. **c, d)** Images from TUG KO HeLa cells co-transfected with IDR1-mCherry (magenta) and Sec23-GFP (green; c) and GMAP210-mNG (green; d). Similar to the full-length protein, the punctate IDR1 structures are separate from ERES and excluded from the cis-Golgi. Data is representative of two independent transfections. **e)** HEK293T cells were transfected with Em-ERGIC53, and the protein was immunoprecipitated using GFP trap agarose beads. In the western blots, cell lysates (input) and eluates from the immunoprecipitated fractions were probed using the GFP antibody (top) and TUG antibody (bottom). Bands corresponding to Em-ERGIC53 are seen in cell lysates and in IP eluates obtained from cells transfected with this plasmid, and this protein is absent from the controls, indicating specificity in the immunoprecipitation. The TUG protein band is only seen in the input fractions and is not co-immunoprecipitated with ERGIC53 using these experimental conditions.

Supplementary Figure 7

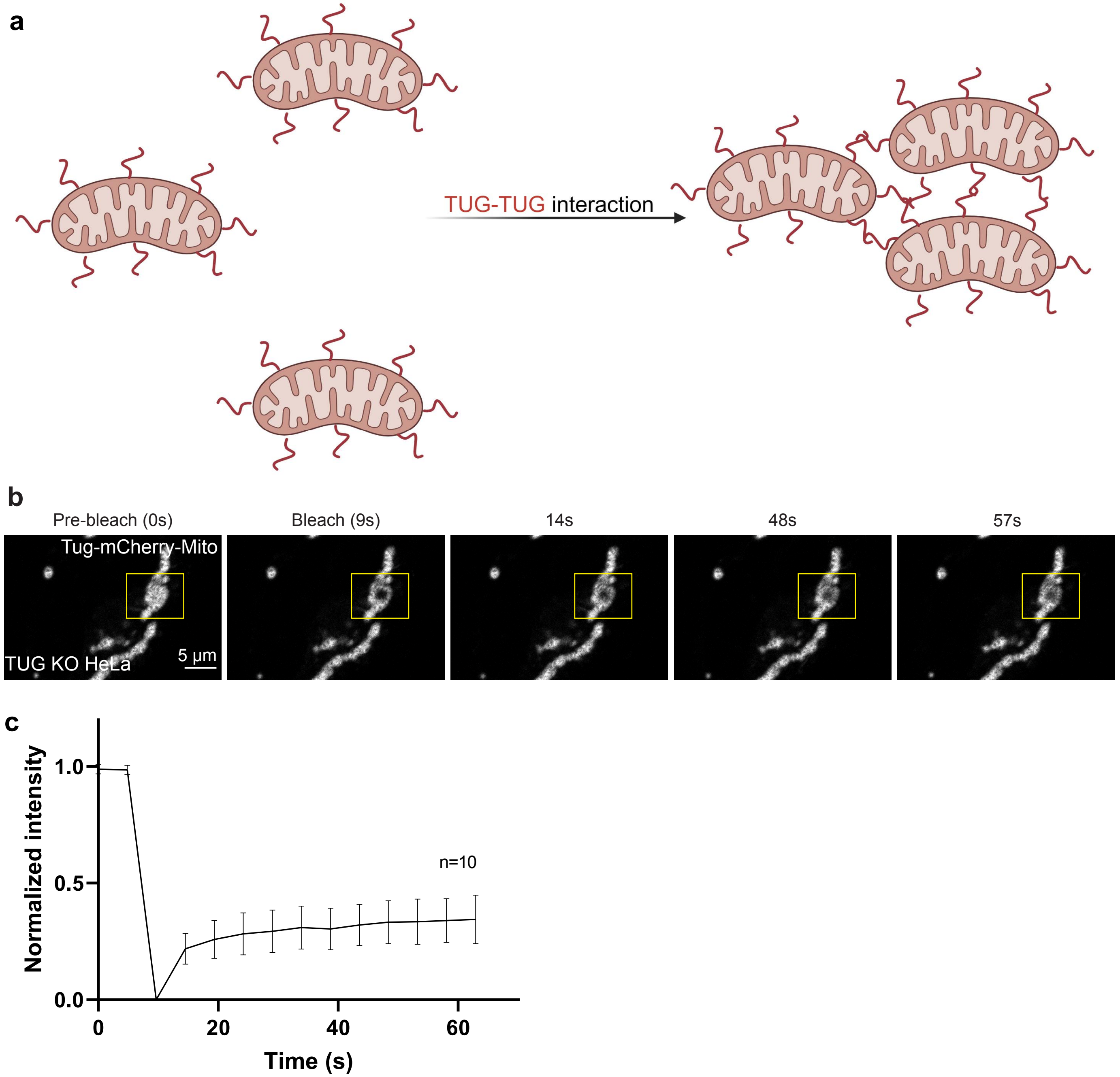

Supplementary Fig. 7 | Targeting of TUG to mitochondria and fluorescence recovery after photobleaching (FRAP) studies

a) Schematic to show mitochondrial clustering driven by TUG protein-protein interaction and oligomerization in trans to explain the phenotypes presented in Fig. 5. **b)** Time series from TUG KO HeLa cells transfected with mitochondrially tethered TUG-mCherry where a small region in the assemblies was bleached (box) followed by time-lapse imaging. **c)** Quantification of normalized intensity as a function of time before and after photobleaching a small region within the clustered assemblies of mitochondria. There is close to 30% recovery within a minute. Note that the majority of the recovery is instantaneous in the first frame after bleaching. Data from N=10 assemblies are represented as mean ± s.d. Created in BioRender. Parchure, A. (2025). https://BioRender.com/9fps5db

Supplementary Figure 8

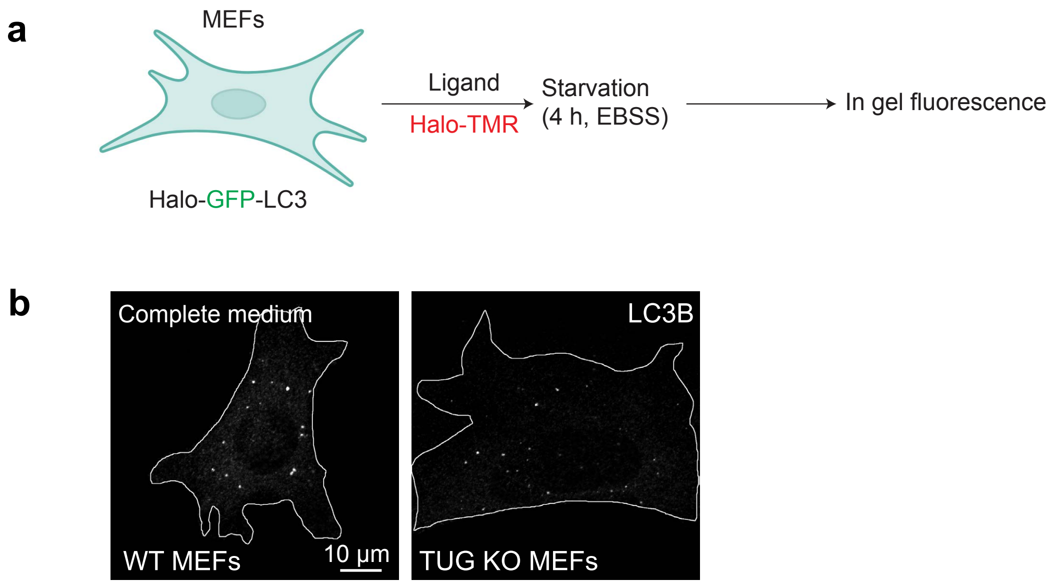

Supplementary Fig. 8 | Supporting data for autophagy studies
a) Schematic the autophagy processing assay using Halo- tagged reporters. WT and TUG KO MEFs expressing Halo- and GFP- tagged LC3 were generated using retroviral expression and FACS sorting. Cells stably expressing the reporter construct were incubated with Halo-TMR for 20 minutes. Cells were then incubated in EBSS for 4 hours to induce autophagy, then harvested, lysed and analyzed by SDS-PAGE. Gels were imaged to assess TMR fluorescence.
b) Images of WT and TUG KO MEFs fixed and stained with antibody against LC3B to observe autophagosomes under basal conditions in cell grown in complete growth medium with 10% serum. Note there are only a few LC3B positive puncta, and these are not significantly different between WT and TUG KO cells. Images are representative of two independent experiments. Created in BioRender. Parchure, A. (2025). https://BioRender.com/2frmni8

Supplementary Figure 9

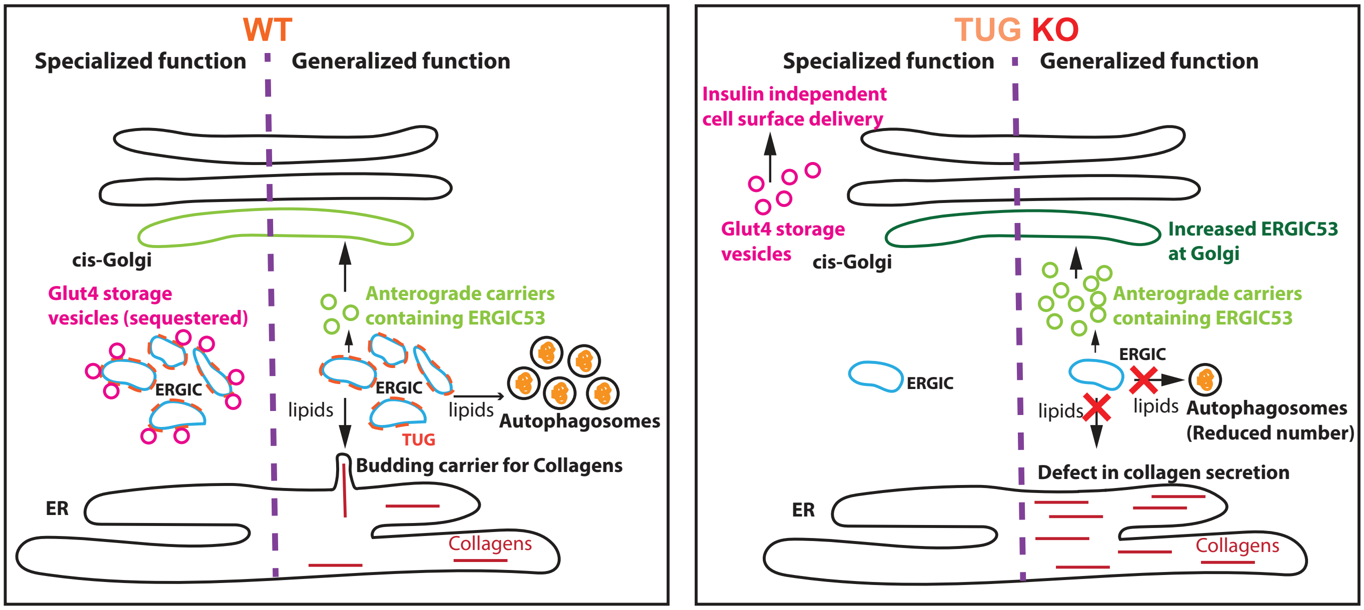

Supplementary Fig. 9 | Model for TUG function at the ERGIC

A model is presented to depict TUG-mediated regulation of the early secretory pathway in specialized and generalized contexts. This model is based on the present work, together with previous studies demonstrating the role of TUG proteins in sequestering GLUT4-containing vesicles (GSVs) in fat and muscle cells. We propose that in specialized fat and muscle cells, TUG protein condensates sequester GSVs at the ERGIC and these vesicles are translocated to the plasma membrane in response to insulin stimulation. In the absence of TUG, there is a constitutive delivery of GLUT4 to the cell surface. A more generalized and ubiquitous function of TUG is to regulate the trafficking from the ERGIC. In the absence of TUG, there is an increased distribution of ERGIC membranes to the cis-Golgi, thus destabilizing the ERGIC. This in turn affects the supply of membranes from the ERGIC for autophagosome biogenesis, and for the formation of large tubular carriers essential for export of bulky cargoes, like collagens, from the ER. Abbreviations: ER, endoplasmic reticulum; ERGIC, endoplasmic reticulum – Golgi intermediate compartment; GSVs, GLUT4 storage vesicles.
