# Supplementary material for: TUG protein acts through a disordered region to organize the early secretory pathway
Source: Nat Commun. 2025 Jul 1;16:5518. doi: 10.1038/s41467-025-60691-8 (PMC12218103; doi:10.1038/s41467-025-60691-8)
Supplement: Supplementary file 2 — Description of Additional Supplementary Information [file 41467_2025_60691_MOESM2_ESM.docx]

**Description of Additional Supplementary Files**

File Name: Supplementary Data 1

Description: Mass spectrometry analysis of secretome from TUG KO MEFs compared to WT control cells.

File Name: Supplementary Data 2

Description: Oligonucleotides used for cloning.
