# Supplementary material for: TUG protein acts through a disordered region to organize the early secretory pathway
Source: Nat Commun. 2025 Jul 1;16:5518. doi: 10.1038/s41467-025-60691-8 (PMC12218103; doi:10.1038/s41467-025-60691-8)
Supplement: Supplementary file 5 — Reporting Summary [file 41467_2025_60691_MOESM5_ESM.pdf]

nature portfolio

Corresponding author(s): Anup Parchure and Jonathan Bogan

Last updated by author(s): Apr 1, 2025

# Reporting Summary

Nature Portfolio wishes to improve the reproducibility of the work that we publish. This form provides structure for consistency and transparency in reporting. For further information on Nature Portfolio policies, see our Editorial Policies and the Editorial Policy Checklist.

## Statistics

For all statistical analyses, confirm that the following items are present in the figure legend, table legend, main text, or Methods section.

| n/a | Confirmed | |
|---|---|---|
| ☐ | ☒ | The exact sample size (*n*) for each experimental group/condition, given as a discrete number and unit of measurement |
| ☐ | ☒ | A statement on whether measurements were taken from distinct samples or whether the same sample was measured repeatedly |
| ☐ | ☒ | The statistical test(s) used AND whether they are one- or two-sided<br>*Only common tests should be described solely by name; describe more complex techniques in the Methods section.* |
| ☒ | ☐ | A description of all covariates tested |
| ☐ | ☒ | A description of any assumptions or corrections, such as tests of normality and adjustment for multiple comparisons |
| ☐ | ☒ | A full description of the statistical parameters including central tendency (e.g. means) or other basic estimates (e.g. regression coefficient) AND variation (e.g. standard deviation) or associated estimates of uncertainty (e.g. confidence intervals) |
| ☐ | ☒ | For null hypothesis testing, the test statistic (e.g. *F*, *t*, *r*) with confidence intervals, effect sizes, degrees of freedom and *P* value noted<br>*Give P values as exact values whenever suitable.* |
| ☒ | ☐ | For Bayesian analysis, information on the choice of priors and Markov chain Monte Carlo settings |
| ☒ | ☐ | For hierarchical and complex designs, identification of the appropriate level for tests and full reporting of outcomes |
| ☒ | ☐ | Estimates of effect sizes (e.g. Cohen's *d*, Pearson's *r*), indicating how they were calculated |

*Our web collection on statistics for biologists contains articles on many of the points above.*

## Software and code

Policy information about availability of computer code

| | |
|---|---|
| Data collection | Zen Black Software and NIS-Elements (Confocal imaging), PONDR, ChemiDoc MP Imaging system (Biorad), ImageQuant LAS400 (Amersham), iTEM imaging software and SerialEM (EM Tomography), Custom code written in Labview (4Pi imaging) |
| Data analysis | Fiji and Imaris 9 (Image analysis), Spectronaut 18 and Qlucore Omics Explorer 3.9 (Mass spec), Vutara SRX 7.0.06 software, Python, PYME-Visualize and MeshLab (4Pi imaging analysis). Figures were prepared using Adobe Photoshop (version 20.0.6) and Illustrator (version 23.1.1; Creative Cloud 2020), and Graphpad Prism (version 9.0) software, Microsoft Excel. |

For manuscripts utilizing custom algorithms or software that are central to the research but not yet described in published literature, software must be made available to editors and reviewers. We strongly encourage code deposition in a community repository (e.g. GitHub). See the Nature Portfolio guidelines for submitting code & software for further information.

## Data

Policy information about availability of data

All manuscripts must include a data availability statement. This statement should provide the following information, where applicable:

- Accession codes, unique identifiers, or web links for publicly available datasets
- A description of any restrictions on data availability
- For clinical datasets or third party data, please ensure that the statement adheres to our policy

All data are available in the manuscript or the supplementary materials.

## Research involving human participants, their data, or biological material

Policy information about studies with human participants or human data. See also policy information about sex, gender (identity/presentation), and sexual orientation and race, ethnicity and racism.

| | |
|---|---|
| Reporting on sex and gender | *Use the terms sex (biological attribute) and gender (shaped by social and cultural circumstances) carefully in order to avoid confusing both terms. Indicate if findings apply to only one sex or gender; describe whether sex and gender were considered in study design; whether sex and/or gender was determined based on self-reporting or assigned and methods used. Provide in the source data disaggregated sex and gender data, where this information has been collected, and if consent has been obtained for sharing of individual-level data; provide overall numbers in this Reporting Summary. Please state if this information has not been collected. Report sex- and gender-based analyses where performed, justify reasons for lack of sex- and gender-based analysis.* |
| Reporting on race, ethnicity, or other socially relevant groupings | *Please specify the socially constructed or socially relevant categorization variable(s) used in your manuscript and explain why they were used. Please note that such variables should not be used as proxies for other socially constructed/relevant variables (for example, race or ethnicity should not be used as a proxy for socioeconomic status). Provide clear definitions of the relevant terms used, how they were provided (by the participants/respondents, the researchers, or third parties), and the method(s) used to classify people into the different categories (e.g. self-report, census or administrative data, social media data, etc.) Please provide details about how you controlled for confounding variables in your analyses.* |
| Population characteristics | *Describe the covariate-relevant population characteristics of the human research participants (e.g. age, genotypic information, past and current diagnosis and treatment categories). If you filled out the behavioural & social sciences study design questions and have nothing to add here, write "See above."* |
| Recruitment | *Describe how participants were recruited. Outline any potential self-selection bias or other biases that may be present and how these are likely to impact results.* |
| Ethics oversight | *Identify the organization(s) that approved the study protocol.* |

Note that full information on the approval of the study protocol must also be provided in the manuscript.

# Field-specific reporting

Please select the one below that is the best fit for your research. If you are not sure, read the appropriate sections before making your selection.

☒ Life sciences ☐ Behavioural & social sciences ☐ Ecological, evolutionary & environmental sciences

For a reference copy of the document with all sections, see nature.com/documents/nr-reporting-summary-flat.pdf

# Life sciences study design

All studies must disclose on these points even when the disclosure is negative.

| | |
|---|---|
| Sample size | Sample sizes were designed to provide 90% power to detect a 1.4 SD difference, based on anticipated variances. |
| Data exclusions | No data was excluded. |
| Replication | Data shown in the manuscript has been all reproducible, including qualitative data and the experiments were performed at least twice and in most instances at least three times. A detailed description of the sample size is provided in the legends . |
| Randomization | Since the study does not involve animals, no randomization was done. |
| Blinding | Blinding was not possible as cells (WT and Knockouts) were differentiated on the basis of differences in Golgi morphology. However, for microscopy based experiments, imaging and analysis were usually done by different individuals and analysis was automated in Imaris, Fiji or using Python codes. |

# Reporting for specific materials, systems and methods

We require information from authors about some types of materials, experimental systems and methods used in many studies. Here, indicate whether each material, system or method listed is relevant to your study. If you are not sure if a list item applies to your research, read the appropriate section before selecting a response.

## Materials & experimental systems

| n/a | Involved in the study |
|---|---|
| ☐ | ☒ Antibodies |
| ☐ | ☒ Eukaryotic cell lines |
| ☒ | ☐ Palaeontology and archaeology |
| ☒ | ☐ Animals and other organisms |
| ☒ | ☐ Clinical data |
| ☒ | ☐ Dual use research of concern |
| ☒ | ☐ Plants |

## Methods

| n/a | Involved in the study |
|---|---|
| ☒ | ☐ ChIP-seq |
| ☒ | ☐ Flow cytometry |
| ☒ | ☐ MRI-based neuroimaging |

# Antibodies

Antibodies used

Antibodies used for immunofluorescence were as follows: GM130 (BD Biosciences; 610822), Sec31A (BD Biosciences; 612350), ERGIC53 (Sigma; E1031), Golgin 97 (Proteintech; 12640-1-AP), P230 (Invitrogen; PA5-87716), LC3A/B (Cell Signalling Technology; D3U4C) Atto 488 conjugated GFP booster (Proteintech; gba488), Atto 595 conjugated RFP booster (Proteintech; rba594). Antibody against KDELr was a kind gift from Dr. James Rothman's laboratory. Fluorophore conjugated secondary antibodies (Alexa 488 and Alexa 568) were obtained from Invitrogen. For 4Pi SMS imaging VHH anti-mouse CF660C (Biotium) and VHH anti-rabbit AF647 (Jackson Immunoresearch) were used. For western blotting, antibodies directed to GFP (Proteintech; P42212) and GAPDH (Millipore Sigma; MAB374) were commercially obtained. The TUG antibody was described previously and is directed to the TUG C-terminal peptide, which is identical in mice and humans. HRP-conjugated secondary antibodies were obtained from Millipore Sigma. Antibody against Sec31A to detect ERES in MEFs was raised in rabbit and is a gift from Dr. Fred Gorelick

Validation

Antibodies were selected carefully upon reviewing data supplied by the manufacturers and based on our own data obtained using these antibodies. Antibodies were validated based on their detection of targets with the predicted molecular weight, predicted expression patterns during development, predicted subcellular location, as well as based on positive and negative controls using overexpression and/or knockdown/knockout in cell lines.

# Eukaryotic cell lines

Policy information about cell lines and Sex and Gender in Research

Cell line source(s)

MEFs, HeLa cells (WT and Knock outs)

Authentication

HeLa cells were not specifically authenticated beyond being obtained from ATCC. MEFs were authenticated based on their genotype.

Mycoplasma contamination

Mycoplasma contamination was tested periodically with Hoechst staining and no contamination was seen.

Commonly misidentified lines
(See ICLAC register)

No commonly misidentified lines noted in the register were used.

# Plants

Seed stocks

*Report on the source of all seed stocks or other plant material used. If applicable, state the seed stock centre and catalogue number. If plant specimens were collected from the field, describe the collection location, date and sampling procedures.*

Novel plant genotypes

*Describe the methods by which all novel plant genotypes were produced. This includes those generated by transgenic approaches, gene editing, chemical/radiation-based mutagenesis and hybridization. For transgenic lines, describe the transformation method, the number of independent lines analyzed and the generation upon which experiments were performed. For gene-edited lines, describe the editor used, the endogenous sequence targeted for editing, the targeting guide RNA sequence (if applicable) and how the editor was applied.*

Authentication

*Describe any authentication procedures for each seed stock used or novel genotype generated. Describe any experiments used to assess the effect of a mutation and, where applicable, how potential secondary effects (e.g. second site T-DNA insertions, mosiacism, off-target gene editing) were examined.*
